# Antagonistic Autoantibodies to Insulin-Like Growth Factor-1 Receptor Associate with Poor Physical Strength

**DOI:** 10.3390/ijms21020463

**Published:** 2020-01-11

**Authors:** Christian Schwiebert, Peter Kühnen, Niels-Peter Becker, Tim Welsink, Theresa Keller, Waldemar B. Minich, Susanna Wiegand, Lutz Schomburg

**Affiliations:** 1 Institute for Experimental Endocrinology, Charité–Universitätsmedizin Berlin, Corporate Member of Freie Universität Berlin, Humboldt-Universität zu Berlin, Berlin Institute of Health, Augustenburger Platz 1, D-13353 Berlin, Germany; Christian.Schwiebert@bruker.com (C.S.); niels-peter.becker@charite.de (N.-P.B.); Tim.Welsink@bruker.com (T.W.); waldemar.minich@charite.de (W.B.M.); 2 Department of Paediatric Endocrinology and Diabetology, Charité–Universitätsmedizin Berlin, Corporate Member of Freie Universität Berlin, Humboldt-Universität zu Berlin, Berlin Institute of Health, Augustenburger Platz 1, D-13353 Berlin, Germany; peter.kuehnen@charite.de (P.K.); susanna.wiegand@charite.de (S.W.); 3 Institute of Biometry and Clinical Epidemiology, Charité–Universitätsmedizin Berlin, Corporate Member of Freie Universität Berlin, Humboldt-Universität zu Berlin, Berlin Institute of Health, Charitéplatz 1, D-10117 Berlin, Germany; theresa.keller@charite.de

**Keywords:** insulin-like growth factor, development, muscle, autoimmunity

## Abstract

Natural autoantibodies to the IGF1 receptor (IGF1R-aAb) have been described in relation to Graves’ ophthalmopathy. Other physiological roles of natural IGF1R-aAb are not known. We hypothesized that IGF1R-aAb may be related to muscle development. Serum samples (*n* = 408) from young overweight subjects (*n* = 143) were collected during a lifestyle intervention study. Anthropometric parameters, along with leptin, IGF1 and IGF1R-aAb concentrations, were analyzed, and the subjects were categorized into positive or negative for IGF1R-aAb. Eleven out of 143 subjects (7.7%) were positive for IGF1R-aAb. Identified IGF1R-aAb were molecularly characterized and showed antagonistic activity in vitro impairing IGF1-mediated IGF1R activation. Mean body weight, height or age were similar between IGF1R-aAb-positive and -negative subjects, but IGF1 concentrations differed. Jumping ability, as well as right and left handgrip strengths, were lower in the IGF1R-aAb-positive as compared to the IGF1R-aAb-negative subjects. We conclude that natural IGF1R-aAb are detectable in apparently healthy subjects and are capable of antagonizing IGF1-dependent IGF1R activation. Moreover, the presence of IGF1R-aAb is associated with poor physical strength. Although the causality of this association is unclear, the data imply a potential influence of IGF1R autoimmunity on muscle development.

## 1. Introduction

Insulin-like growth factor 1 receptor (IGF1R) constitutes a central signaling molecule of the growth hormone (GH) axis, controlling, e.g., bone and muscle development. The active receptor resides as a heterotetrameric (α2β2) transmembrane glycoprotein in the plasma membrane of target cells. Both insulin-like growth factor 1 (IGF1) and IGF2, as well as supraphysiological concentrations of insulin, can serve as positive ligands, all activating the same intracellular downstream cascade that involves the ligand-dependent autophosphorylation of tyrosyl residues in the cytoplasmic domain of IGF1R [1]. IGF1R activation has direct effects on gene expression, protein synthesis, carbohydrate and lipid metabolism, cell proliferation and survival [2]. Studies with transgenic mouse models have highlighted its central importance for muscle and bone growth [3]. Mutations in the genes encoding either IGF1R or IGF1 cause pre- and postnatal growth restrictions and developmental delay [4]. The pathogenic spectrum of the affected patients depends on the particular type and site of mutation and its structural consequences [5]. Besides controlling regular growth, strength and metabolism, IGF1R-dependent signalling is of major importance for cancer initiation and progression [6].

For unknown reasons, autoantibodies (aAb) against endocrine receptors develop in a subset of human subjects. The most prominent example is the thyrotropin receptor (TSHR). Both blocking and stimulating TSHR-aAb are described as modulators of the thyroid axis, causing a hypo- or hyperthyroid phenotype [7,8]. The stimulation of the TSHR by TSHR-aAb is the underlying cause of Graves’ disease (GD), and associated with risk of Graves’ ophthalmopathy (GO) [9]. Interestingly, IGF1R-aAb have also been implicated in GD, potentially synergizing with TSHR-aAb in the pathogenesis of GO by stimulating retro-orbital tissue expansion and synthesis of extracellular matrix, thereby causing eyeball proptosis [10,11,12]. This hypothesis is supported by cell culture experiments, indicating a synergistic cross-talk between both receptors [13,14]. However, a direct quantification of natural IGF1R-aAb failed to detect a difference between GO patients and controls [15]. In order to test for the potential physiological relevance of natural IGF1R-aAb, we characterized IGF1R-aAb in vitro and analysed measures of strength in a cohort of young, overweight subjects participating in a multimodal lifestyle intervention study aiming to reduce and maintain body weight [16]. The rationale for choosing this cohort study was mainly based on its excellent clinical characterization, the young age of the subjects enrolled, ensuring a lack of comorbidities and age-related diseases, and the availability of longitudinal samples, allowing an analysis of IGF1R-aAb stability over time. Our data indicate that IGF1R-aAb impair IGF1 signalling and are associated with relatively poor physical strength.

## 2. Results

### 2.1. Prevalence of IGF1R-aAb in Overweight Young Subjects

**Figure 1:**
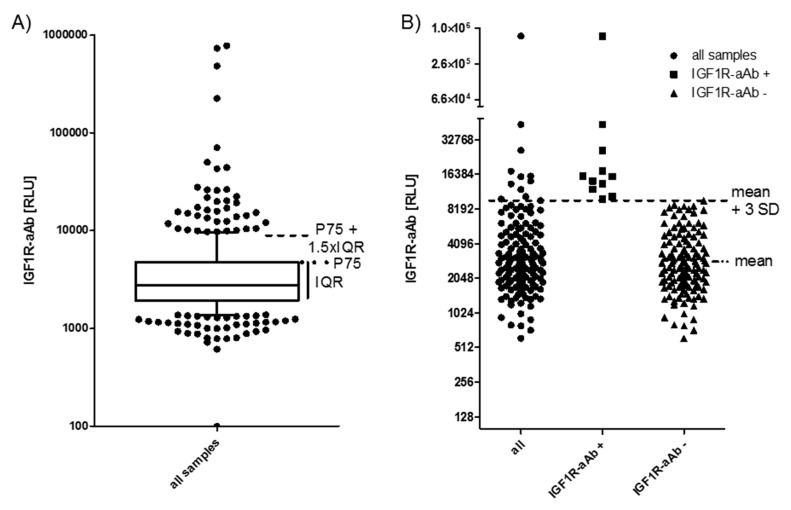
IGF1-Receptor autoantibodies (IGF1R-aAb) in serum of the study subjects. (**A**) Sera from the full collection of samples (*n* = 408) were analyzed for IGF1R-aAb, and relative light units (RLU) were recorded. The outlier criterion of P75 plus 1.5 times the interquartile range (P75 + 1.5 × IQR) was applied to identify samples with positive IGF1R-aAb. (**B**) Analysis of the study population at time point T-3 (three months before study start). This analysis used the outlier criterion mean plus three standard deviations (mean + 3 SD). Irrespective of outlier criterion used (**A** or **B**), both analyses identified the same 11 out of 143 subjects as positive for natural IGF1R-aAb.

All of the available serum samples (*n* = 408) were analysed for the presence of IGF1R-aAb. The signals obtained (relative light units (RLU)) showed a skewed distribution (Figure 1A). Several readings were extraordinarily high (please note the logarithmic scale of the axis), indicating the presence of considerable amounts of IGF1R-aAb. As the threshold for defining positivity, two outlier criteria were applied: either the sum of the 75th percentile value (P75) plus 1.5 times the interquartile range (IQR) of all samples (P75 + 1.5 × IQR) (Figure 1A), or the sum of the mean value within the normal distribution (excluding the highest 10% of samples) plus three times the standard deviation (mean + 3SD) (Figure 1B). The procedures indicated a threshold value of 9057 RLU (4786 RLU + 1.5 × 2847 RLU, Figure 1A), and 8952 RLU (3186 RLU + 3 × 1922 RLU, Figure 1B), respectively.

Applying these two threshold criteria to our data yields a prevalence of 11.3%–11.5% of IGF1R-aAb-positive samples (Figure 1A,B). For the analysis of the potential relevance of the IGF1R-aAb for the clinical parameters, the subjects were classified according to the results at the earliest timepoint available, i.e., at enrolment into the study (T-3). Irrespective of the criterion used, the same eleven subjects were classified as positive for IGF1R-aAb (Figure 1B).

### 2.2. Stability of IGF1R-aAb Concentrations during the Study Period

**Figure 2:**
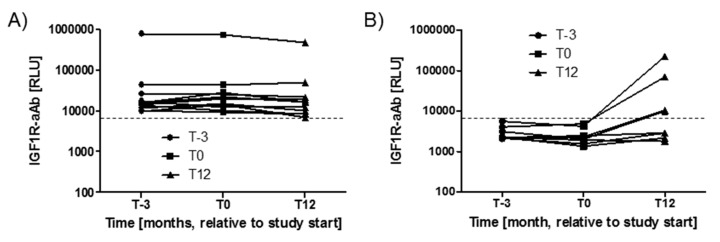
Variations in IGF1R-Ab concentrations with time. The serum samples drawn from the study participants covered a time period of 15 months (enrolment, T-3; study start, T0; and study end, T12). (**A**) The majority of positive samples remained positive during the full study period, indicating stable IGF1R-aAb concentrations over time. (**B**) Four samples developed natural IGF1R-aAb de novo during the study. The signal strengths of these samples are shown in comparison to four constantly negative samples. The threshold for positive IGF1R-aAb is indicated by the thin broken lines.

Variations in IGF1R-aAb concentrations with time during the study period were analysed next. The three timepoints available from the majority of study subjects spanned 15 months, i.e., from three months before intervention (T-3), via start (T0), to the end of the twelve months study period (T12). During this time, the majority of subjects with positive IGF1R-aAb at T-3 (*n* = 11) remained positive for IGF1R-aAb, with notably very little change in signal strength (Figure 2A). Four subjects from the group of IGF1R-aAb negative subjects developed de novo natural IGF1R-aAb concentrations, and their samples taken at T12 surpassed the thresholds to positivity (Figure 2B), whereas the remaining subjects stayed below the threshold, i.e., were negative for natural IGF1R-aAb.

### 2.3. Molecular Characterization of IGF1R-Ab

**Figure 3:**
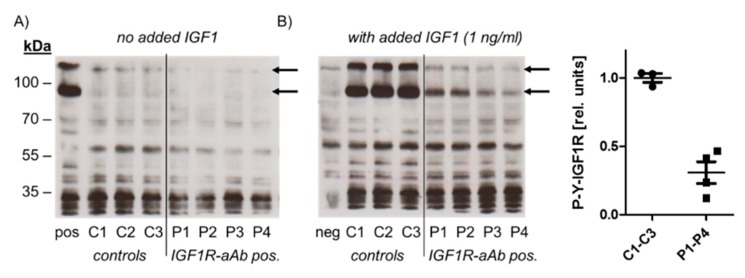
Molecular analysis of the biological activity of the IGF1R-aAb. Immunoglobulins were isolated from IGF1R-aAb negative (controls, C1–C3) and the four most positive (P1–P4) serum samples, respectively, along with an IGF1-stimulated (pos) and non-stimulated negative control (neg) sample. Cell homogenates were analyzed by Western blot for receptor activation using antibodies against Tyr-phosphorylated IGF1R (P-Y-IGF1R). (**A**) In the absence of the added ligand IGF1, autophosphorylation was at basal level irrespective of the added immunoglobulins. (**B**) IGF1R autophosphorylation was induced in the presence of added IGF1 in cells co-incubated with immunoglobulins from control sera, but only faint signals were detected from IGF1-stimulated cells in the presence of immunoglobulins from IGF1R-aAb positive sera. The arrows indicate the two major bands of phosphorylated IGF1R in HepG2 cells, and the graphic depicts the band intensities of P-Y-IGF1R after IGF1 stimulation.

In order to test for a potential biological activity of IGF1R-aAb, immunoglobulins of three control (C1–C3) and four sera with positive IGF1R-aAb (P1–P4) were isolated and analysed (Figure 3).

To this end, HepG2 cells were incubated with the immunoglobulins isolated from control and positive sera, respectively, in the absence or presence of ligand (IGF1, added to a final concentration of 1 ng/mL). Western blot analysis with antiserum specific for Tyr-phosphorylated IGF1R was used to detect IGF1R activation and the effects of IGF1R-aAb on IGF1-dependent signalling. In the absence of added ligand, autophosphorylation of IGF1R was at a low basal level both in the cells incubated with immunoglobulins from control and from IGF1R-aAb-positive sera (Figure 3A). Stimulation of the cells with IGF1 caused strong IGF1R autophosphorylation in the presence of control immunoglobulins, but not in the presence of immunoglobulins isolated from IGF1R-aAb-positive sera, indicating an antagonistic activity of IGF1R-aAb against IGF1 signaling (Figure 3B).

### 2.4. Comparison of Anthropometric Parameters in Relation to IGF1R-Ab

**Figure 4:**
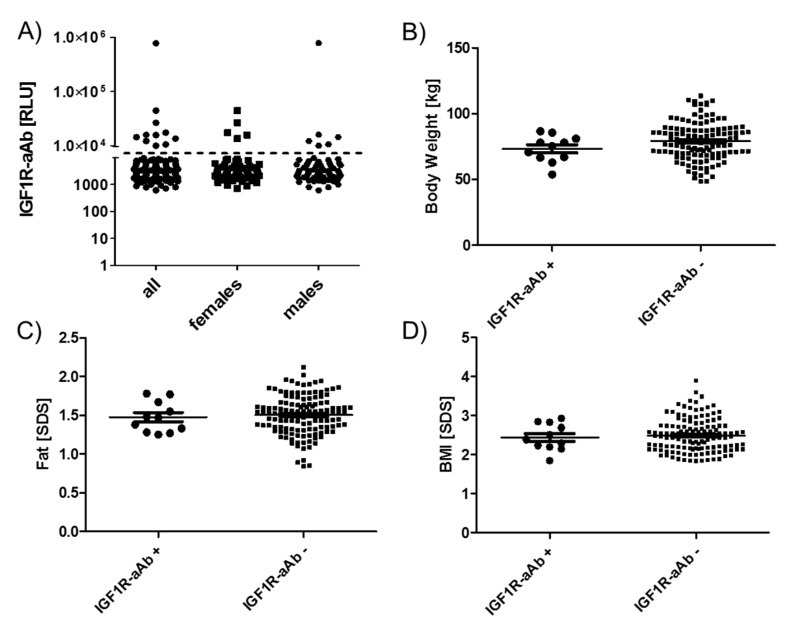
Anthropometric parameters of IGF1R-Ab-positive and -negative subjects. Subjects were categorized as IGF1R-aAb-positive or -negative, as explained in Figure 1. The two resulting groups of subjects, with or without IGF1R-aAb, were not different with respect to (**A**) sex distribution, (**B**) body weight, (**C**) body fat, or (**D**) BMI. Analysis by two-sided non-parametric U Mann–Whitney Test.

The group of eleven subjects identified as IGF1R-aAb-positive showed no sex bias, i.e., six males and five females were positive, from a total of 76 females and 67 males in the full cohort (Figure 4A). Similarly, age distribution and mean age in the groups of IGF1R-aAb-positive and -negative subjects showed no differences (12.7 +/- 2.0 vs. 13.6 +/- 1.8 years at T-3).

As the subjects participated in a weight reduction and maintenance trial, body weight (Figure 4B), age-adapted fat (Figure 4C) and age-adapted BMI (Figure 4D) were high in comparison to reference values, but not different between the groups of IGF1R-aAb-positive and -negative subjects. The results of body composition analysis by bioelectrical impedance analysis (bia) also revealed no significant differences between natural IGF1R-aAb-positive versus -negative subjects (not shown).

### 2.5. Comparison of Endocrine and Physical Strength Parameters in Relation to IGF1R-aAb

**Figure 5:**
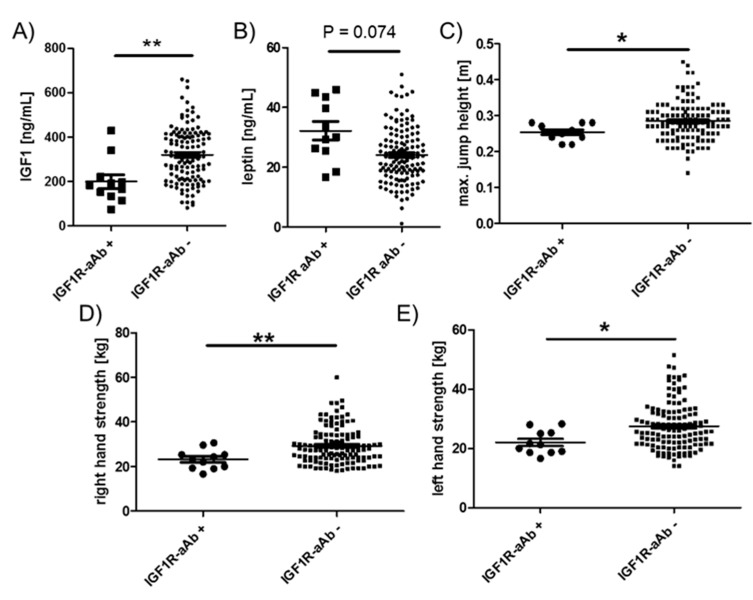
Comparison of clinical parameters related to physical strength. Study subjects were categorized as IGF1R-aAb-positive or -negative, as explained in Figure 1. The two resulting groups of subjects with or without IGF1R-aAb were compared with respect to (**A**) IGF1 concentrations, (**B**) leptin concentrations, (**C**) maximal jump height, as well as (**D**) right hand, and (**E**) left hand grip strengths. Analysis by two-sided non-parametric U Mann–Whitney Test; * *p* < 0.05, ** *p* < 0.01.

Among the endocrine parameters, a lower average concentration of circulating IGF1 in the group of IGF1R-aAb-positive, as compared to the IGF1R-aAb-negative, subjects was observed (181.9 (133.4, 220.9) vs. 316.8 (211.4, 410.2) ng/mL, Figure 5A). In parallel, leptin concentrations differed in the inverse direction, i.e., leptin was slightly elevated in the IGF1R-aAb-positive subjects (30.1 (25.5, 43.4) vs. 23.3 (18.1, 29.9) ng/mL, Figure 5B). IGF1R-aAb-positive subjects showed relatively poor jumping abilities with little inter-individual variation (25.0 (24.0, 28.0) vs. 28.0 (25.0, 31.5) cm, Figure 5C). Along this line, right handgrip strength (23.3 (19.3, 25.3) vs. 27.6 (23.6, 34.1) kg, Figure 5D) and left handgrip strength (21.3 (18.6, 25.3) vs. 26.0 (21.6, 32.1) kg, Figure 5E) were lower in IGF1R-aAb-positive subjects as compared to the group of IGF1R-aAb-negative subjects. Again, inter-individual variability was particularly low within the IGF1R-aAb-positive group as compared to the group of IGF1R-aAb-negative subjects.

## 3. Discussion

In this explorative study on the molecular nature and potential role of natural IGF1R-aAb, we present evidence for an antagonistic activity and a relatively frequent presence of natural IGF1R-aAb in healthy young subjects. The results obtained are in agreement with the hypothesis that natural IGF1R-aAb are of physiological relevance and associated with meaningful endocrine parameters of metabolism and physical strength, potentially limiting regular muscle development.

The prevalence of IGF1R-aAb in the subjects analysed in this study was 7.7%, i.e., slightly lower than the 10–11% observed in our study with adult GO patients [15]. This difference may be disease-, age- and sex-related, as the prevalence of autoantibodies is typically high in autoimmune patients, higher in adults than in children, and generally higher in females than in males [17]. Recently, an independent study using a novel commercially available IGF1R-aAb assay detection kit reported a prevalence of around 25% in patients [18]. The detection method was based on an IGF1R protein preparation, immobilized directly to a solid surface and detecting the number of immunoglobulins binding to the recombinant protein. In our experience, this type of assay design is difficult to conduct for membrane proteins. Due to the low concentrations of natural aAb and the presence of other membrane constituents, the signal-to-noise ratio is typically high, as reported before, e.g., during TSHR-aAb assay development [19,20,21], or in relation to aAb to cardiac receptors of the G-protein coupled receptor family [22,23]. Nevertheless, additional studies comparing different detection methods are needed to highlight the particular strengths and weaknesses of different approaches and test their potential suitability for the diagnosis of aAb-related conditions.

Besides the two methods for quantifying IGF1R-aAb mentioned above, there are also biological assays for receptor-specific aAb that quantify biochemical effects. Such bioassays typically use purified immunoglobulins, and have been described for studying, e.g., aAb to the TSHR [24] or aAb to cardiac receptors [25]. In GD, the hyperthyroid phenotype results from the specific agonistic binding of stimulating aAb to the TSHR [7,8]. Similarly, stimulating aAb to cardiac receptors are implicated in Chagas’ disease [26,27]. However, antagonizing aAb are also described, e.g., in type B insulin resistance, where aAb to the insulin receptor impairs insulin effects, causing a form of diabetes that is refractory to therapeutic insulin application [28]. In our initial study on IGF1R aAb, we isolated immunoglobulins from adult patients with GO and tested their effects on IGF1 signaling and the proliferation of MCF7 breast cancer cells in vitro [15]. In agreement with the data presented here, IGF1-induced autophosphorylation of IGF1R was specifically suppressed by immunoglobulins isolated from IGF1R aAb-positive sera, indicating antagonistic effects on IGF1 signalling in vitro. Such antagonistic activities of IGF1R-aAb would be compatible to the clinical findings observed in this study, i.e., reduced physical strength in combination with decreased IGF1 concentrations, which may reflect a suppressed GH axis causing poor IGF1-dependent muscle development. The reduced jumping ability and handgrip strengths would also be compatible with an antagonistic action of the IGF1R-aAb on IGF1-promoted anabolic effects on muscle [29]. Notably, the differences in left hand grip strength were slightly more pronounced than the right hand differences, indicating a developmental, not habitual, origin of this difference. The reduced IGF1 concentration in the presence of IGF1R-aAb is reminiscent of findings in children with IGF1R mutations [30,31]; however, there are also reports from subjects with defective IGF1R signaling where increased circulating IGF1 levels have been observed [5], and additional studies are needed to resolve the underlying mechanism.

A particular strength of our study is given by the relatively large collection of samples from young, healthy, but overweight subjects, the intensive clinical characterization of several muscle-related parameters, the availability of longitudinal samples for monitoring IGF1RaAb concentrations over time, and the in vitro characterization of the antagonistic nature of IGF1R-aAb. Moreover, the relatively and unexpectedly strong associations are in line with the biological knowledge of the relevance of the GH axis for muscle development, and the findings appear plausible in view of the antagonistic activity elicited by IGF1R-aAb.

There are some notable limitations. Firstly, the number of IGF1R-aAb-positive subjects was relatively small in comparison to IGF1R-aAb-free subjects. Secondly, no normal-weight children were analysed, making it difficult to deduce the potential relevance of IGF1R-aAb for obesity. This shortcoming is due to ethical constraints not allowing serum collection from healthy children for research purposes. Thirdly, our study was observational and explorative, and not designed to identify potential causal relationships, which remain to be tested in prospective studies.

## 4. Materials and Methods

### 4.1. Recombinant Expression of IGF1R as Luciferase Fusion Protein

Stable HEK 293 cells expressing high levels of recombinant IGF1R as a fusion protein with firefly luciferase (IGF1R-Luc) were grown in DMEM supplemented with 10% FBS, and used for the IGF1R-aAb assay, as described previously [15]. Briefly, confluent cells were harvested into PBS and lysed in resuspension buffer (20 mM HEPES-NaOH, pH 7.5, 50 mM NaCl, 1% Triton X-100, and 10% glycerol). The suspension was cleared by centrifugation at 5000 rpm, and the supernatant was collected and stored at −70 C, and aliquots were thawed when needed and used for the measurements.

### 4.2. Molecular Characterization of IGF1R Autoantibodies

Protein A sepharose was used to purify immunoglobulins from a set of IGF1R-aAb-positive and -negative serum samples, respectively. After centrifugation of the pellets and extensive washing with PBS, immunoglobulins were eluted with citric acid (pH 2.5), neutralized with HEPES (pH 8.0) until reaching pH 7.0, concentrated and transferred into DMEM/F12 medium by gel filtration, as described earlier [15]. Isolated immunoglobulins were added to hepatocellular liver carcinoma cells (HepG2) for 1 h in serum-free medium in the presence or absence of 1 ng/mL (f. c.) of recombinant human IGF1 (OriGene Technologies GmbH, Herford, Germany). Cells were then lysed in Triton X-100 (2% f. c.) containing lysis buffer, proteins were separated by 10% SDS-PAGE, blotted onto nitrocellulose membrane, and activated IGF1R was detected by anti-IGF1R (Tyr1165/Tyr1166) antibody (Novus Biologicals, Edinburgh, United Kingdom). Signals were quantified by image analysis software (ImageJ, NIH, Bethesda, MD, USA).

### 4.3. Serum Samples from Children Enrolled in the Intervention Trial

Serum samples (*n* = 408) from obese children and adolescents (*n* = 143) participating in an intervention trial for body weight reduction (“MAINTAIN”) were collected in the Department of Paediatric Endocrinology and Diabetology, Charité—Universitätsmedizin Berlin, at different time points, as described earlier [16]. The study had been conducted in accordance with the guidelines in the Declaration of Helsinki, it was approved by the ethical review committee of Charité—Universitätsmedizin Berlin (EA2/015/09, 8 April 2009) and had been registered at ClinicalTrials (NCT00850629, 17 February 2009). Informed consent of the subjects and/or one or both parent(s) was obtained prior to study entry. The samples analysed had been obtained three months before (T-3), at study entry (T0) and after study completion (T12). Samples were analysed by personnel blinded to the clinical characteristics.

### 4.4. Quantification of IGF1R Autoantibodies

IGF1R-aAb were quantified by a precipitation test as described previously [15]. Briefly, a fusion protein of full-length IGF1R-Luc was incubated with 10 µL of serum overnight at 4 °C. Formed complexes were precipitated with protein A sepharose (Sigma-Aldrich, St. Louis, MO, USA), and pellets were washed three times with washing buffer (10 mM Tris-HCl, pH 7.5, 60 mM NaCl, 0.02% Tween 20). Luciferase activities were measured in a luminometer (Mitras, Berthold Technologies GmbH, Bad Wildbad, Germany), and results were recorded as relative light units (RLU).

### 4.5. Anthropometric Analyses

Three months before lifestyle intervention (T-3), a set of anthropometric variables was analysed from all participants, including age, weight, height, grip strength, jumping ability and waist circumference [16]. A blood sample was taken and endocrine parameters were determined by the local diagnostics provider conducting routine clinical measurements (Labor Berlin—Charité Vivantes GmbH). The same parameters were determined again at the start of the intervention (T0) and twelve months later (T12).

### 4.6. Statistical Analysis

Statistical analysis was performed using GraphPad Prism v4.0 (GraphPad Software Inc., San Diego, CA, USA) and with SAS version 9.4 (SAS Institute, Cary, NC, USA). Data are presented as median and IQR, with the exception of age and sex. To test for differences between IGF1R-aAb-positive and IGF1R-aAb-negative participants, we used two-sided non-parametric U Mann–Whitney Tests. The *p*-values may not be interpreted as confirmative, as all analyses were considered exploratory and not adjusted for multiple testing.

## 5. Conclusions

We conclude that a subset of children express IGF1R-aAbs that are associated with reduced physical strength. While this association may seem unfortunate to the IGF1R-aAb-positive subjects, potentially impairing the anabolic activities of IGF1 and hindering full muscle development, reduced activity in the GH axis is also reported to provide health benefits. Several studies in experimental models and humans with Laron syndrome have clearly highlighted that reduced cancer and diabetes risks and higher odds for longevity can be expected under such conditions [32]. It is hoped that these effects prevail in the children analysed.

## Abbreviations

BRCA1	BReast CAncer 1
BRCA2	BReast CAncer 2
PALB2	PArtner and Localizer of BRCA2
PDAC	pancreatic ductal adenocarcinoma
TMAs	Tissue microarrays
IHC	immunohistochemistry
OS	Overall survival
FANCN	Fanconi anemia complementation
TCGA	The Cancer Genome Atlas
GSEA	gene set enrichment analysis
CIs	confidence intervals
HR	hazard ratio
T	primary tumor site
N	regional lymph node involvement
NES	normalized enrichment score
EMT	epithelial to mesenchymal transition
